# Gradient mesoporosity in hierarchical ZIF-8 by temperature-modulated soft-templating

**DOI:** 10.1039/d5sc05218a

**Published:** 2025-10-24

**Authors:** Keisuke Shirasaki, Yingji Zhao, Norman C.-R. Chen, Xiangyang Liu, Yusuke Asakura, Kevin C.-W. Wu, Yusuke Yamauchi

**Affiliations:** a Department of Materials Process Engineering, Graduate School of Engineering, Nagoya University Nagoya Japan zhao.yingji.n2@f.mail.nagoya-u.ac.jp; b Molecular Science and Technology Program, Taiwan International Graduate Program, Academia Sinica Taipei 10617 Taiwan; c International Graduate Program of Molecular Science and Technology (NTU-MST), National Taiwan University Taipei 10617 Taiwan; d Department of Chemical Engineering, National Taiwan University Taipei 10617 Taiwan; e Department of Chemical Engineering and Materials Science, Yuan Ze University Zhongli District Taoyuan 32003 Taiwan; f Australian Institute for Bioengineering and Nanotechnology (AIBN), School of Chemical Engineering, The University of Queensland Brisbane Queensland Australia y.yamauchi@uq.edu.au; g Department of Chemical and Biomolecular Engineering, Yonsei University 50 Yonsei-ro, Seodaemun-gu Seoul 03722 South Korea

## Abstract

Engineering hierarchical porosity in metal–organic frameworks (MOFs) is critical for improving mass transport and expanding their utility in catalysis, separation, and adsorption. We report a temperature-controlled soft-templating method for precise tuning of mesopore size in zeolitic imidazolate framework-8 (ZIF-8), employing a single amphiphilic block copolymer as the structure-directing agent. Systematic variation of the synthesis temperature yields a distinctive gradient mesoporous architecture, characterized by a progressive increase in mesopore diameter from the particle interior to the exterior. This strategy enables continuous control over mesostructure without the need for additional swelling agents or template exchange. The resulting trimodal porous ZIF-8 exhibits significantly enhanced structural accessibility and diffusion characteristics, providing a versatile platform for future studies in catalysis and molecular adsorption.

## Abstract 2

Synthesis of hierarchical porous ZIF-8 with tunable mesopore sizes achieved by varying the synthesis temperature.

## Graphical abstract

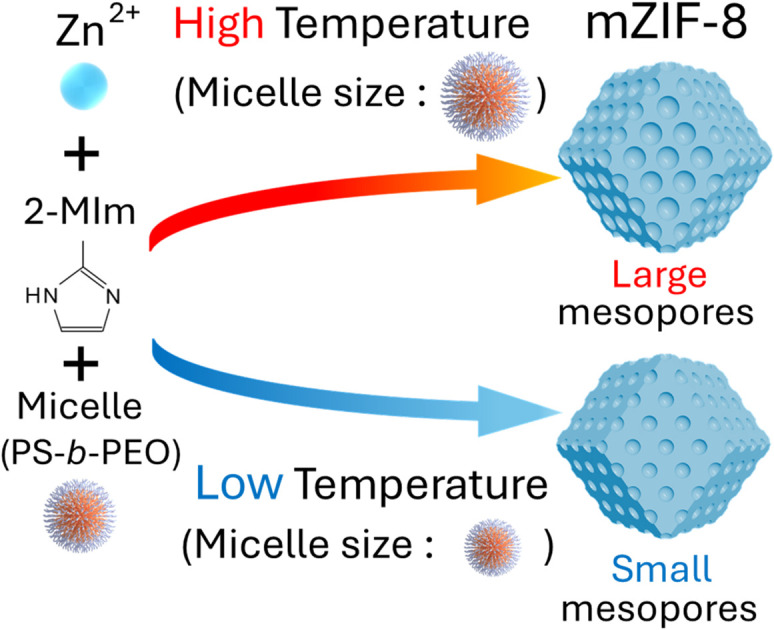

## Introduction

Metal–organic frameworks (MOFs) and porous coordination polymers (PCPs) are crystalline microporous materials composed of inorganic metal centers coordinated to organic ligands.^1^ These materials feature highly tunable structural characteristics—including controllable pore sizes, diverse morphologies, and adjustable surface polarities—achievable through rational selection of metal–ligand combinations.^2,3^ Owing to their exceptionally high surface area and robust thermal stability, MOFs, particularly zeolitic imidazolate frameworks (ZIFs),^4,5^ have shown considerable promise in diverse applications such as gas storage,^6,7^ catalysis,^8,9^ separation,^10,11^ and water purification.^12,13^ However, their intrinsic microporous structure, with pores typically smaller than 2 nm, often severely restricts diffusion of larger molecules, limiting broader practical applicability.^14–16^ Although nanoparticle downsizing can partially address this issue by shortening diffusion pathways, internal micropores remain largely inaccessible to bulky guest molecules.

To effectively overcome these diffusion limitations, researchers have increasingly focused on introducing hierarchical porosity into microporous MOFs through templating strategies. For instance, incorporation of macropores (≥50 nm), for example, using polystyrene (PS) templates, has been demonstrated to enhance molecular accessibility.^17,18^ However, macropores contribute only minimally to host–guest interactions and typically do not lead to significant improvements in surface area. In contrast, mesoporous MOFs, characterized by pores ranging from 2 to 50 nm, offer an optimal balance between efficient diffusion and substantial surface interactions, making them particularly advantageous for catalysis,^19^ adsorption,^20^ and sensing^21^ applications. Among various approaches, the self-assembly templating strategy has been recognized as an effective method to introduce mesoporosity into MOFs,^22^ although the resulting mesopores are often non-uniform compared with those obtained *via* the soft-templating method. Recent advancements in soft-templating using surfactants have successfully produced hierarchical micro/mesoporous MOFs,^23–29^ exemplified initially by mesoporous UiO-66,^23^ with subsequent refinements achieving monodisperse, single-crystalline particles.^24,25^

**Scheme 1:**
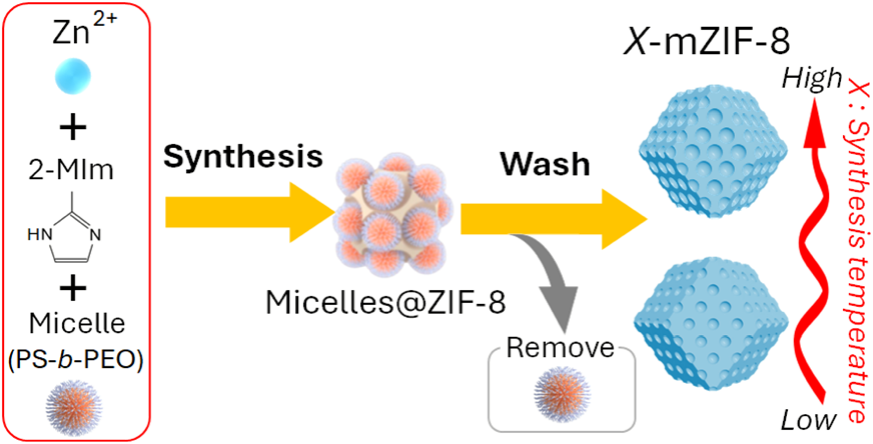
Synthesis scheme of *X*-mZIF-8.

While soft-templating methods are attractive due to their simplicity and mild conditions, conventional mesopore-size modulation predominantly relies on varying the molecular weights of hydrophobic segments within amphiphilic block copolymer templates or swelling the hydrophobic core of micelles.^30,31^ Developing alternative methods for precise mesopore-size tuning utilizing a single block copolymer type would significantly enhance the versatility, scalability, and controllability of mesoporous MOF synthesis. Furthermore, multimodal mesoporous MOFs^32^—particularly those featuring bimodal mesopore architectures—exhibit superior physicochemical properties, including increased catalytic site accessibility and improved spatiotemporal control over reactions.^33,34^ However, achieving precise thermodynamic control over pore-size distribution, uniformity, and interconnectivity remains an ongoing synthetic challenge. In this study, we report a temperature-mediated strategy for precisely controlling mesopore size in ZIF-8 using a fixed soft-template (Scheme 1). Notably, modulation of the synthesis temperature during crystal growth results in a unique hierarchical architecture, characterized by a gradient increase in mesopore size from the particle core to its surface.

## Results and discussion

**Fig. 1:**
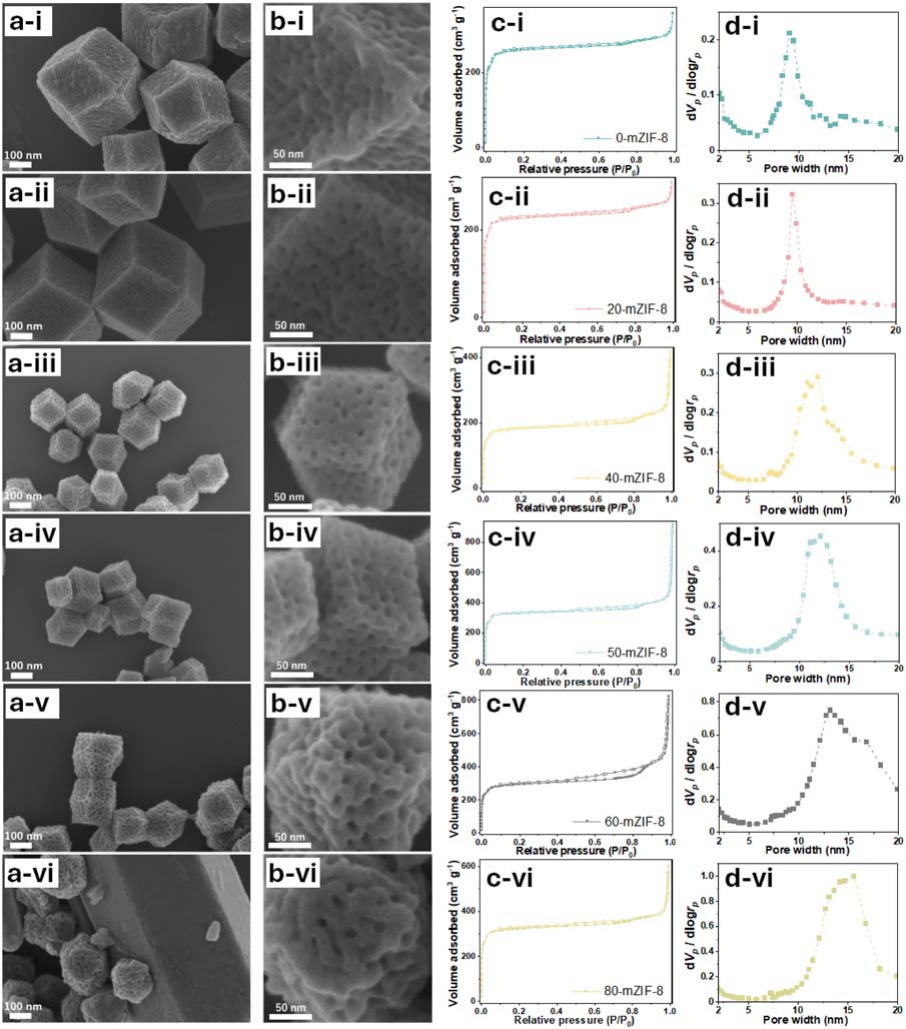
(a) SEM images, (b) magnified SEM images, (c) nitrogen adsorption–desorption isotherms, and (d) BJH pore size distributions of *X*-mZIF-8, where *X* represents the synthesis temperature (°C): (i) 0 °C, (ii) 20 °C, (iii) 40 °C, (iv) 50 °C, (v) 60 °C, and (vi) 80 °C.

Micro–meso-hierarchical porous ZIF-8 (mZIF-8) is synthesized following a previously reported procedure,^25,35^ with synthesis temperatures ranging from 0 °C to 80 °C. Zn^2+^ ions are reacted with 2-methylimidazole (2-MIm) in the presence of polystyrene-*block*-poly(ethylene oxide) (PS_5k_-*b*-PEO_2.5k_) micelles, which served as soft-templates for mesopore formation. In this soft-templating system, the hydrophobic polystyrene (PS) segments form the micellar cores, while the hydrophilic poly(ethylene oxide) (PEO) chains interact with Zn^2+^ ions and 2-MIm in the surrounding solution. During the crystallization of ZIF-8, Zn^2+^ ions coordinated to the PEO chains promote framework growth around the micellar aggregates, and subsequent removal of the copolymer yields ordered mesopores. Thus, the mesostructure formation is governed by the self-assembly of block copolymer micelles and their interfacial coordination with inorganic precursors.^25^ The successful synthesis of *X*-mZIF-8 is confirmed by scanning electron microscopy (SEM), X-ray diffraction (XRD), and nitrogen adsorption–desorption isotherms. SEM images reveal that mesopore size increases with rising synthesis temperature, and the resulting ZIF-8 particles exhibit a well-defined rhombic dodecahedral morphology, indicating that crystal growth have reached a stable phase (Fig. 1a and b).^36^ Given that micelle behavior is influenced by parameters such as temperature, ion concentration, and solvent conditions, the observed increase in mesopore size is likely due to temperature-induced changes in micelle structure and dynamics.^37^ In addition, SEM observation shows that the particle size of *X*-mZIF-8 gradually decreases with increasing synthesis temperature. Since particle size is governed by the balance between nucleation and crystal growth,^38,39^ the observed decrease in particle size with increasing temperature is likely due to a higher nucleation rate and reduced crystal growth under elevated temperatures. Interestingly, SEM images of 80-mZIF-8 reveal the presence of pillar-shaped crystals without mesoporous structures as byproducts (Fig. 1a-vi and S1). The absence of mesopores in the pillar-shaped crystals may result from the polymer functioning as a morphology-directing agent rather than as a micellar template, as polymers are known to occasionally play such a role under specific synthesis conditions.^40^

**Fig. 2:**
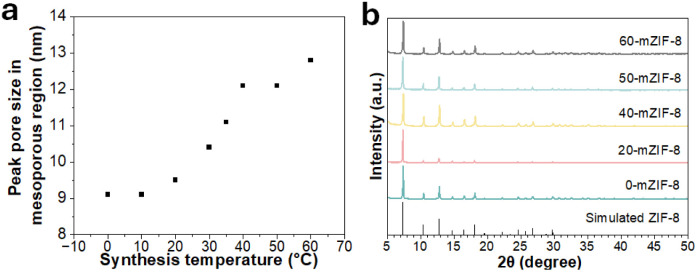
(a) Synthesis temperature *vs.* peak pore size in the mesoporous region of *X*-mZIF-8, and (b) XRD spectra of *X*-mZIF-8.

To confirm the crystal structure of the synthesized *X*-mZIF-8 samples, X-ray diffraction (XRD) analysis was performed. The XRD patterns reveals that all *X*-mZIF-8 samples exhibit diffraction peaks consistent with those of simulated ZIF-8, indicating the successful formation of the ZIF-8 framework and preservation of its primary crystal structure across the range of synthesis temperatures (Fig. 2b). Notably, the 80-mZIF-8 sample also shows an identical diffraction pattern to simulated ZIF-8, confirming that the pillar-shaped crystals correspond to the ZIF-8 phase.

To confirm the porosity of *X*-mZIF-8, nitrogen adsorption–desorption measurements and SEM-based pore size analysis were conducted for samples synthesized at temperatures ranging from 0 °C to 60 °C (Fig. 1c, d and S2). The resulting isotherms are indicative of a hierarchical micro–mesoporous structure (Fig. 1c).^41^ The micropores originate from the inherent crystal structure of ZIF-8, while the mesopores are attributed to the presence of the soft-template used during synthesis. A plot of synthesis temperature *versus* peak pore size in the mesoporous region, as determined from the Barrett–Joyner–Halenda (BJH) pore size distribution, reveals that the peak mesopore size increases from approximately 9 nm to 13 nm as the synthesis temperature is raised from 0 °C to 60 °C (Fig. 2a). In addition, the temperature-dependent increase in pore size is also confirmed by SEM-based pore size analysis (Fig. S2). To further investigate the effect of the micellar template on the intrinsic microporous structure, the non-local density functional theory (NLDFT) method was applied to 0-mZIF-8, 60-mZIF-8, and conventional ZIF-8 (Fig. S3). The NLDFT results indicate that all the samples exhibit similar pore size distributions in the microporous region, suggesting that the presence of the polymer template does not alter the intrinsic microporous framework of ZIF-8.

Based on these measurements, it is confirmed that mZIF-8 is successfully synthesized at temperatures between 0 °C and 60 °C. Accordingly, *X*-mZIF-8 samples synthesized within this temperature range were further characterized using transmission electron microscopy (TEM), energy-dispersive X-ray spectroscopy (EDS), and Fourier-transform infrared spectroscopy (FT-IR), and their chemical stability is further examined in a range of solvents.

TEM images clearly demonstrate an increase in mesopore size with rising synthesis temperature and reveal the presence of internal pore structures (Fig. S4). EDS elemental mapping confirms the uniform distribution of 2-methylimidazole (2-MIm) and Zn throughout the crystals (Fig. S5). Specifically, the uniform distribution of nitrogen and carbon indicates homogeneous incorporation of the organic linker, while the even distribution of zinc confirms the uniformity of the metal sites.

The chemical structure of the synthesized ZIFs is characterized by FT-IR spectroscopy, and the presence of residual polymer is also examined (Fig. S6). Characteristic absorption bands are observed in the ranges of 950–1350 cm^−1^, ∼1585 cm^−1^, 1350–1500 cm^−1^, 2890–3199 cm^−1^, and 3200–3600 cm^−1^. These bands correspond to the out-of-plane ring bending, in-plane ring bending, and C=N stretching, entire ring vibrations, C–H stretching (both aromatic and aliphatic), and N–H stretching vibrations of the 2-methylimidazole ligand, respectively.^42^ A weak absorption peak around 2700 cm^−1^, which is absent in conventional ZIF-8 but present in PS-*b*-PEO, is observed in all mZIF-8 samples, suggesting the possible presence of trace polymer residues even after repeated washing, although most of the polymer was successfully removed.

The chemical stability of 60-mZIF-8 and conventional ZIF-8 was further evaluated in various solvents to investigate the framework stability (Fig. S7). Both the samples retain their structural integrity after immersion in acetone, acetonitrile, and toluene for 3 h. However, exposure to water leads to partial structural degradation in both samples. Notably, 60-mZIF-8 exhibits higher water stability than conventional ZIF-8, although some degree of damage is still observed. This enhanced stability may be attributed to the presence of trace PS chains from the residual PS-*b*-PEO template, which could impart partial hydrophobic protection to the framework.

In addition, dynamic light scattering (DLS) measurements of micelle solutions at 0 °C and 60 °C confirm that the average micelle size increases with rising temperature (Fig. S8), suggesting that the temperature-dependent growth of micelles likely contributes to the formation of larger mesopores in the resulting mZIF structures.

To investigate the effect of the zinc salt on mesoporosity, a series of mZIF-8 samples was synthesized using zinc acetate instead of zinc nitrate, while keeping all other synthesis conditions, including the crystal growth time (4 h), unchanged. These samples are denoted as *X*-OAc-mZIF-8, where *X* represents the synthesis temperature. The mesoporous structures are evaluated by SEM imaging and nitrogen adsorption–desorption measurements.

**Fig. 3:**
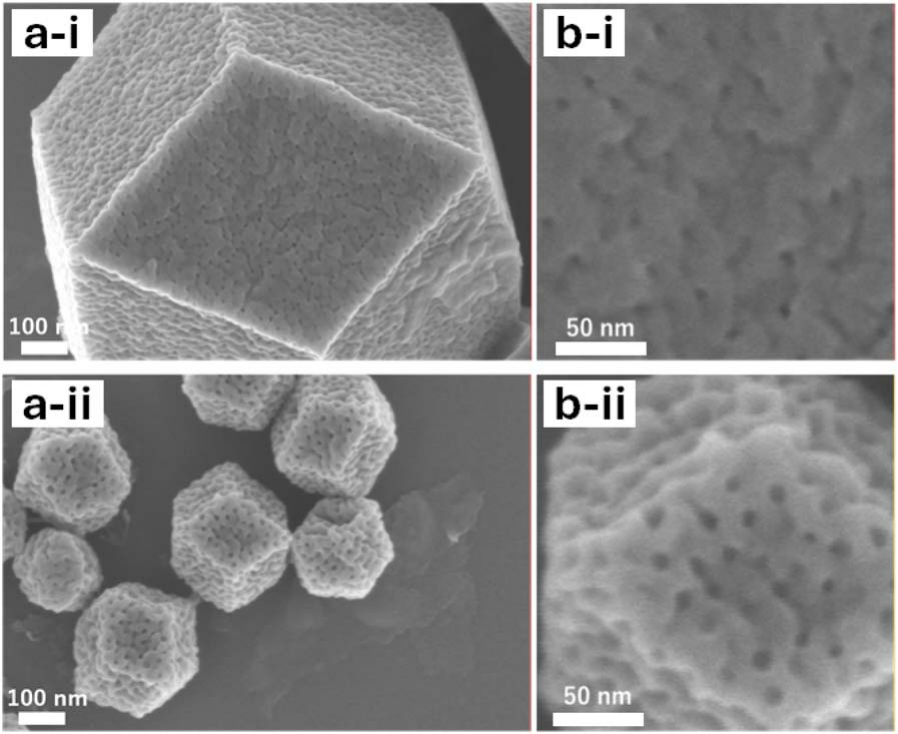
(a) SEM images and (b) magnified SEM images of *X*-OAc-mZIF-8, where *X* represents the synthesis temperature (°C): (i) 0 °C and (ii) 50 °C.

SEM images of *X*-OAc-mZIF-8 reveal that the mesopore size increased gradually with rising synthesis temperature, similar to the trend observed for *X*-mZIF-8 (Fig. 3). Furthermore, the mesoporous structure of *X*-OAc-mZIF-8 appears rougher than that of *X*-mZIF-8. This rougher mesostructure may be attributed to an increased number of framework defects, possibly caused by the chelating effect of acetate ions on zinc ions during synthesis.

Nitrogen adsorption–desorption analysis further supports these findings. The BJH pore size distributions, calculated from the adsorption branches of the isotherms, also show an increase in mesopore size with higher synthesis temperatures. Moreover, the pore size distributions of *X*-OAc-mZIF-8 are broader and less uniform compared to those of *X*-mZIF-8 (Fig. S9), consistent with the SEM observations. To investigate whether the presence of acetate ions influences mesoporosity, sodium acetate (NaOAc) was added during the synthesis of mZIF-8. As shown in Fig. S10, the BJH pore size distribution shows progressively broader with increasing NaOAc concentration. This result indicates that acetate ions play a key role in modulating the mesostructure, likely by interacting with zinc ions and introducing additional framework irregularities during crystal growth.

To verify whether the crystal structure or chemical bonding is altered by using zinc acetate instead of zinc nitrate,^43^ XRD and XPS measurements are performed (Fig. S11 and S12). The XRD pattern confirms that *X*-OAc-mZIF-8 exhibits the same crystal structure as simulated ZIF-8, indicating that mZIF-8 can also be synthesized using Zn(OAc)_2_ in place of Zn(NO_3_)_2_. These results demonstrate that the choice of zinc salt significantly influences the mesoporous architecture of the synthesized mZIF-8. Considering that the size of mesopores can be controlled by changing synthesis temperature, it is possible that we can introduce small mesopores as a core part, and big mesopores as a shell part by changing temperature during crystal growth.

By applying the above protocol with zinc acetate as the metal source, a micro–meso–meso trimodal mesoporous ZIF-8 (TMP-OAc-mZIF-8) was synthesized *via* a two-step growth mechanism. In the first step, mZIF-8 was prepared at 0 °C for 2 h, which is 2 h shorter than the typical growth time (4 h). This sample is referred to as 0/2 h-OAc-mZIF-8. Subsequently, a secondary mZIF-8 layer was grown at 50 °C for an additional 10 min to form the TMP-OAc-mZIF-8 structure.

**Fig. 4:**
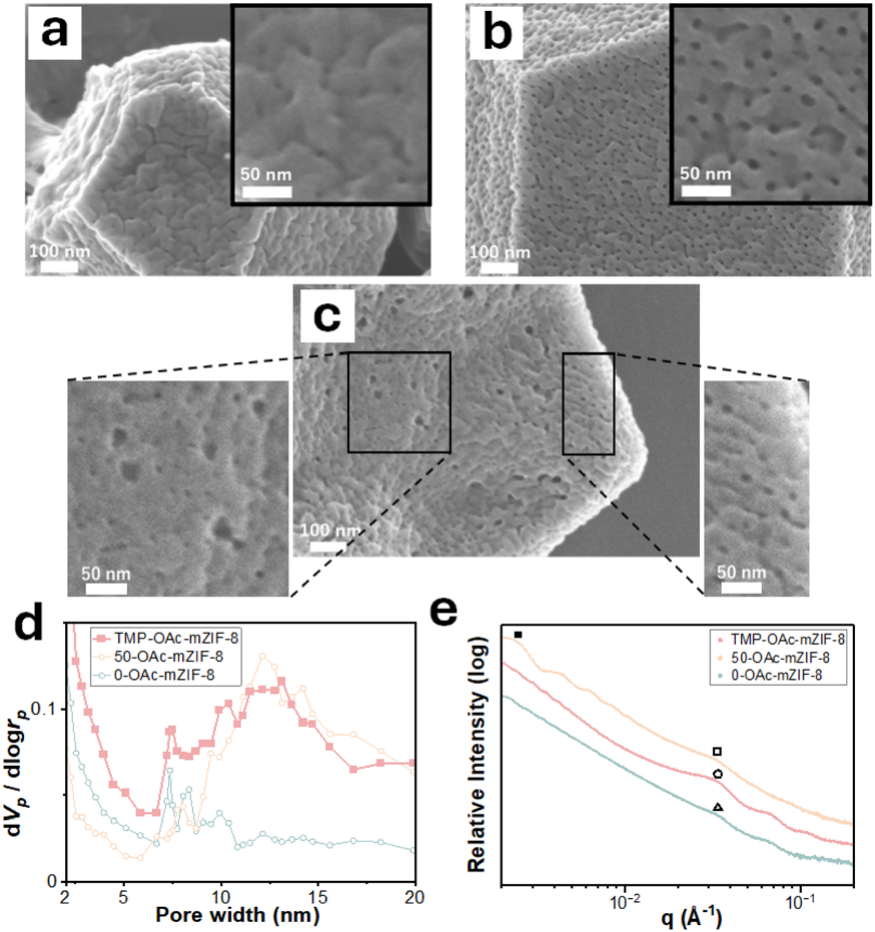
SEM images of (a) 0/2 h-mZIF-8 and (b) TMP-mZIF-8. (c) Cross-sectional SEM image of TMP-mZIF-8, showing magnified views of the core and shell regions. (d) BJH pore size distributions and (e) SAXS 1D profiles of TMP-mZIF-8, 50-mZIF-8, and 0-mZIF-8.

The SEM image of 0/2 h-OAc-mZIF-8 shows similar size of mesoporous structure with 0-OAc-mZIF-8 (Fig. 3b and 4a), smaller mesoporous structure than 50-OAc-mZIF-8, suggesting that the size of mesoporous structure is corresponding to the synthesis temperature even the crystal growth is in progress. The SEM image of TMP-OAc-mZIF-8 shows that big mesoporous structure like 50-OAc-mZIF-8 on the surface (Fig. 4b), indicating that the crystal growth phase for 10 min at 50 °C create the second layer, which show bigger mesoporous structure than synthesized at 0 °C.

The formation of the micro–meso–meso hierarchical pore structure is confirmed by particle size distribution analysis *via* DLS, cross-sectional SEM imaging, and nitrogen adsorption–desorption measurements. DLS results shows that the particle size of 0/2 h-OAc-mZIF-8 is approximately 700 nm, while that of TMP-OAc-mZIF-8 is approximately 1200 nm (Fig. S13), indicating that the 10 minutes crystal growth at 50 °C contributed an additional ∼500 nm in size, corresponding to the formation of an outer shell layer.

Cross-sectional SEM images and the TEM image of TMP-OAc-mZIF-8 reveal two distinct mesoporous domains: the inner region exhibits smaller mesopores, while the outer region contains larger mesopores, consistent with the synthesis temperatures of 0 °C and 50 °C, respectively (Fig. 4c and S14). The approximate diameter of the inner region and thicknesses of the outer layer are 400 nm and 200 nm, respectively (Fig. S15), which is in good agreement with the particle size increase observed by DLS.

In addition, BJH pore size distribution curves calculated from the adsorption branch of nitrogen isotherms display a unique bimodal peak in the mesoporous region (Fig. 4d and S16). This bimodal profile closely resembles the superimposed distributions of 0-OAc-mZIF-8 and 50-OAc-mZIF-8, supporting the coexistence of two distinct mesopore sizes within a single particle. The presence of micropores derived from the ZIF-8 framework is also confirmed by the adsorption isotherm (Fig. S16).

To further verify the mesoporous architecture of TMP-OAc-mZIF-8, small-angle X-ray scattering (SAXS) measurements were performed. The 0-OAc-mZIF-8 sample exhibits a characteristic scattering peak at a q-value of 0.035 Å^−1^ (△ mark), while the 50-OAc-mZIF-8 sample shows peaks at 0.033 Å^−1^ (□ mark) and 0.0025 Å^−1^ (■ mark). Both 0-OAc-mZIF-8 and 50-OAc-mZIF-8 display similar peaks around 0.034 Å^−1^, indicating that the inter-pore distances in the two samples are comparable, despite differences in individual mesopore sizes. The peak at 0.0025 Å^−1^ corresponds to a particle size of approximately 250 nm,^44^ which is consistent with the SEM observations (Fig. 3a-ii). Furthermore, the SAXS 1D profile of TMP-OAc-mZIF-8 exhibits the same peak as those of the two reference samples (○ mark), suggesting that the inter-pore distances are unchanged even when the synthesis temperature is altered during crystal growth (Fig. 4e).

## Conclusions

In this study, we have successfully synthesized mesoporous ZIF-8 (mZIF-8) using a soft-templating method and demonstrated that the mesopore size can be effectively controlled by adjusting the synthesis temperature. At lower temperatures (*e.g.*, 0 °C), the resulting mesopores are relatively small, with diameters around 9 nm. In contrast, higher synthesis temperatures (*e.g.*, 60 °C) yield larger mesopores, approximately 12 nm in diameter. Furthermore, we achieve the design of a micro–meso–meso three-phase hierarchical porous structure within a single ZIF-8 particle simply by varying the synthesis temperature during crystal growth. This work provides new insights into soft-templated MOF synthesis, particularly for tailoring hierarchical pore architectures without the need for complex procedures or multiple templates.

## Author contributions

Y. Zhao conceived the project. K. Shirasaki performed the experiments, analysed the data and drafted the manuscript. N. Chen, X. Liu, Y. Asakura, and K. Wu helped collect some of the experimental data. Y. Zhao and Y. Yamauchi supervised the research.

## Conflicts of interest

There are no conflicts to declare.

## Supplementary Material

SC-016-D5SC05218A-s001

## Data Availability

The data supporting this article are included in the SI and are also available from the corresponding author upon reasonable request. Supplementary information: experimental section, additional characteristic data. See DOI: https://doi.org/10.1039/d5sc05218a.
