# Supplementary material for: Gradient mesoporosity in hierarchical ZIF-8 by temperature-modulated soft-templating
Source: Chem Sci. 2025 Oct 24;16(47):22742–7. doi: 10.1039/d5sc05218a (PMC12576841; doi:10.1039/d5sc05218a)
Supplement: SC-016-D5SC05218A-s001 [file SC-016-D5SC05218A-s001.pdf]

Supplementary Information (SI) for Chemical Science.
This journal is © The Royal Society of Chemistry 2025

Supporting Information

Gradient Mesoporosity in Hierarchical ZIF-8 by Temperature-Modulated Soft-Templating

Keisuke Shirasaki,[a] Yingji Zhao,*[a] Norman C.-R. Chen,[a,b,c] Xiangyang Liu,[a] Yusuke Asakura,[a] Kevin C.-W. Wu,[b,c,d,e] and Yusuke Yamauchi*[a,f,g]

a. *Department of Materials Process Engineering, Graduate school of engineering, Nagoya University, Nagoya, Japan. E-mail: zhao.yingji.n2@f.mail.nagoya-u.ac.jp*

b. *Molecular Science and Technology Program, Taiwan International Graduate Program, Academia Sinica, Taipei 10617, Taiwan*

c. *International Graduate Program of Molecular Science and Technology (NTU-MST), National Taiwan University, Taipei 10617, Taiwan*

d. *Department of Chemical Engineering, National Taiwan University, Taipei 10617, Taiwan*

e. *Department of Chemical Engineering and Materials Science, Yuan Ze University, Zhongli District, Taoyuan 32003, Taiwan*

f. *Australian Institute for Bioengineering and Nanotechnology (AIBN) and School of Chemical Engineering, The University of Queensland, Brisbane, Queensland, Australia. E-mail: y.yamauchi@uq.edu.au*

g. *Department of Chemical and Biomolecular Engineering, Yonsei University, 50 Yonsei-ro, Seodaemun-gu, Seoul 03722, South Korea.*

Experimental Section/Methods

Chemicals

All reagents were purchased from commercial suppliers and used without further purification. Zinc nitrate hexahydrate (99 %), zinc acetate Dihydroxide (99 %), and acetone (99 %) were supplied by Fujifilm Wako Pure Chemical Co., Ltd. (Japan). Ethanol (99.5 %) was obtained from Kishida Chemical Co., Ltd. (Japan). 2-Methylimidazole (2-MIm, 99 %) was purchased from Sigma-Aldrich. The block copolymer PS_{5k}-*b*-$PEO_{2.5k}$ (Product ID: P40298-SEO) was purchased from Polymer Source Inc.

Synthesis of X-mZIF-8 and X-OAc-mZIF-8

Initially, 9 mg of PS_{5k}-*b*-$PEO_{2.5k}$ was dissolved in 100 μL of tetrahydrofuran (THF). Subsequently, 2 mL of an aqueous solution of 2-MIm was added under constant stirring. After stirring for 10 minutes at *X* °C, 2 mL of a 40 mM aqueous solution of $Zn(NO_3)_2{\cdot}6H_2O$ or $Zn(OAc)_2{\cdot}2H_2O$ was quickly injected into the mixture. The resulting solution was stirred for 5 minutes and then left undisturbed at *X* °C for 4 hours.

The resulting precipitate was collected by centrifugation at 12,000 rpm for 5 minutes, followed by four sequential washing steps using ethanol and acetone. Each washing step involved the addition of solvent to the centrifuge tube, ultrasonic treatment for 10 minutes, and subsequent centrifugation at 12,000 rpm for 5 minutes. Ethanol was used for the first and fourth washes, and acetone was used for the second and third. Finally, the product was dried overnight at 60 °C to yield the target material (denoted as *X*-mZIF-8 or *X*-OAc-mZIF-8, where *X* indicates the synthesis temperature).

Synthesis of conventional ZIF-8

All procedures were identical to those described above, except that PS-*b*-PEO was not used during synthesis.

Synthesis of mZIF-8 with NaOAc

All procedures were performed as described above, except for the composition of the zinc precursor solution. Instead of using a 40 mM $Zn(NO_3)_2{\cdot}6H_2O$ solution, a mixed solution containing 40 mM $Zn(NO_3)_2{\cdot}6H_2O$ and X mM NaOAc (X = 0, 10, 40) was used. The synthesis was conducted at room temperature.

Synthesis of TMP-OAc-mZIF-8

All procedures were the same as described above, except for the crystal growth step. After injecting the 40 mM aqueous solution of $Zn(OAc)_2$, the solution was maintained at 0 °C for 2 hours, followed by incubation at 50 °C for 10 minutes

Chemical stability test

For the chemical stability test, 4 mg of Conventional ZIF-8 or 60-mZIF-8 was dispersed in 4 mL of acetonitrile, acetone, toluene, or water. The suspensions were ultrasonicated briefly and then left at room temperature for 3 hours.

Characterization Techniques

XRD patterns were obtained using a Rigaku SmartLab diffractometer operated at 40 kV and 30 mA with Cu Kα radiation. The scan rate was set to 5° min-1. SEM analysis was conducted using a ZEISS GeminiSEM 560 at an accelerating voltage of 2 kV. Nitrogen adsorption–desorption isotherms were measured using a MicrotracBEL BEL-mini analyzer. The samples were degassed at 100 °C for 10 h prior to measurement. The pore size distribution was evaluated from the adsorption branch using the BJH method. Transmission Electron Microscopy and Energy Dispersive X-ray Spectroscopy (TEM-EDS) analysis was performed with a JEOL JEM-2100 Plus operating at 200 kV. Dynamic light scattering (DLS) measurements were performed using an Otsuka ELSZ-2000 instrument to determine the particle size distribution. X-ray photoelectron spectroscopy (XPS) was conducted using a PHI Quantes spectrometer (ULVAC-PHI Inc.). Ultraviolet diffuse reflectance spectroscopy (UV-DRS) spectra were recorded on a JASCO V-770 spectrophotometer. Fourier transform infrared (FT-IR) spectra were obtained using a JASCO FT/IR 4X. Small-angle X-ray scattering (SAXS) measurements were carried out at beamline TLS-13A1 (BioSAXS) of the NSRRC, Taiwan, using 15 keV photon energy and two Eiger detectors (X 1M and X 9M).

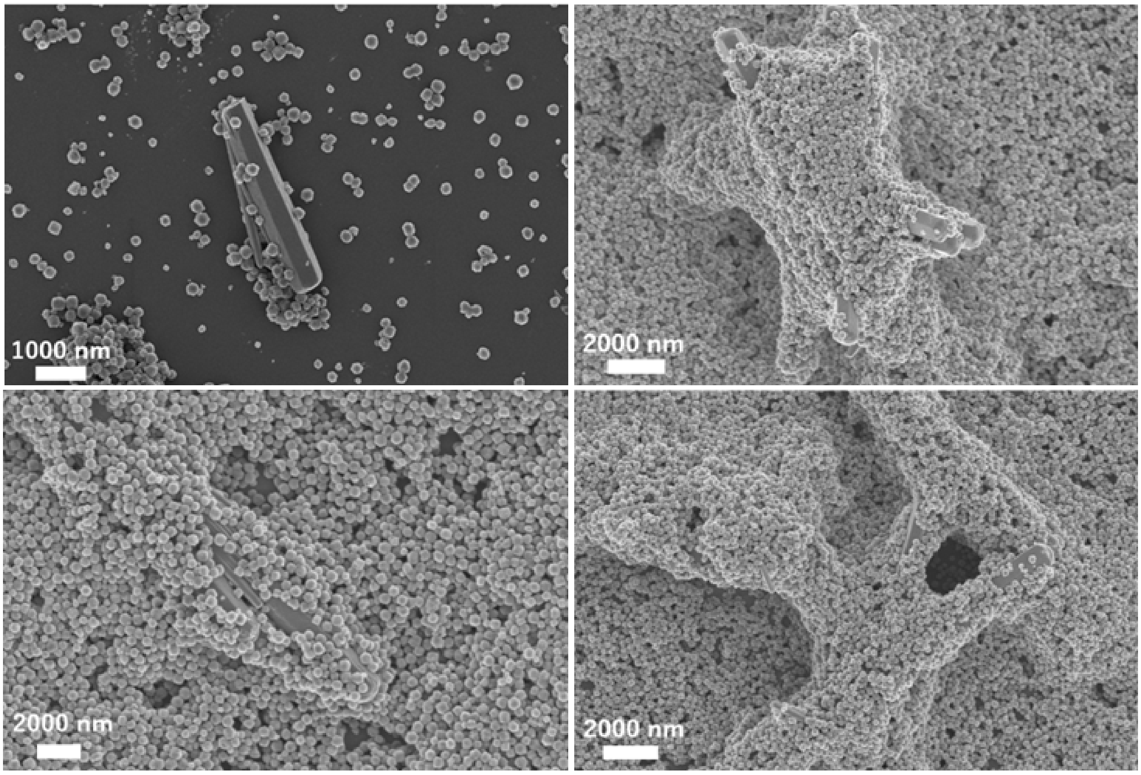

Figure S1 Low-magnification SEM images of the byproduct of 80-mZIF-8

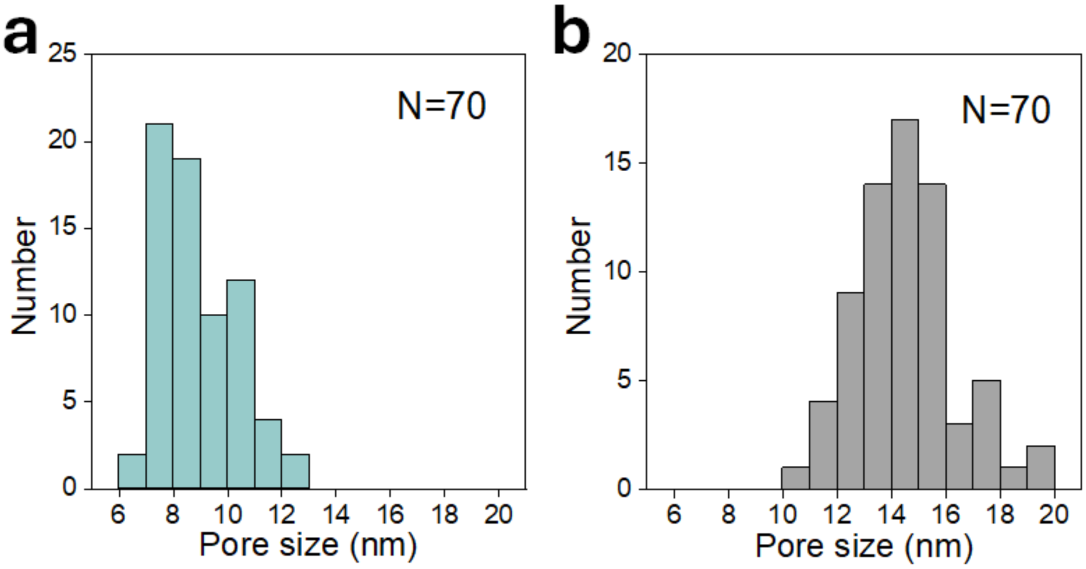

Figure S2 Pore size distribution of (a) 0-mZIF-8 and (b) 60-mZIF-8 calculated from SEM images.

Note for **Figure S2** : SEM-based pore size distributions were determined by approximating each pore on non-tilted surfaces as an ellipse using ImageJ, calculating the equivalent circle diameters, and plotting their distribution.

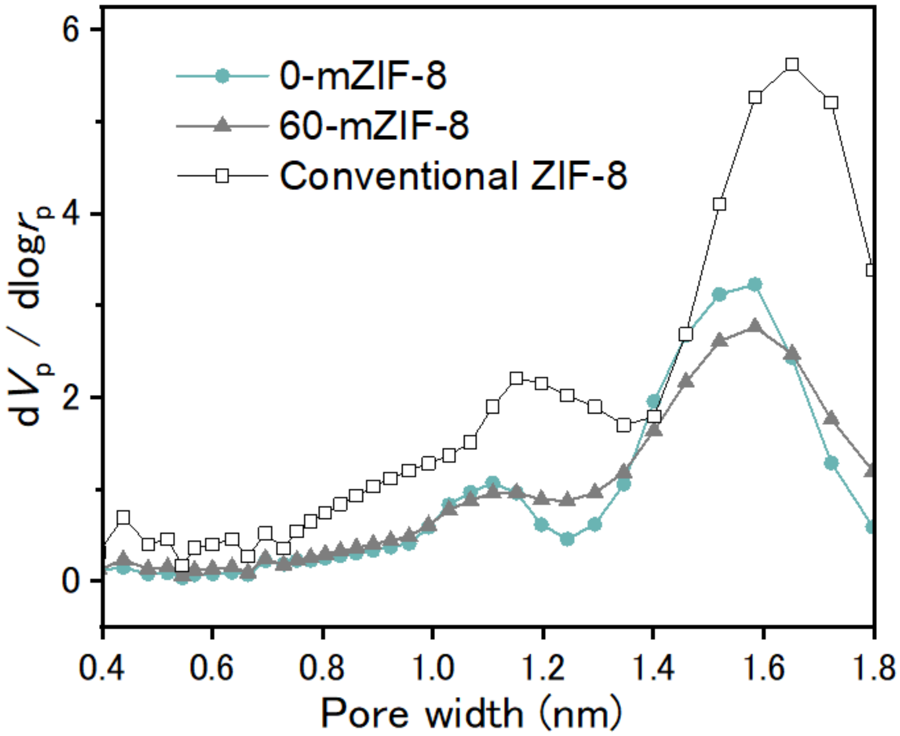

Figure S3 Micropore size distribution curves of 0-mZIF-8, 60-mZIF-8, and conventional ZIF-8, obtained using the NLDFT method.

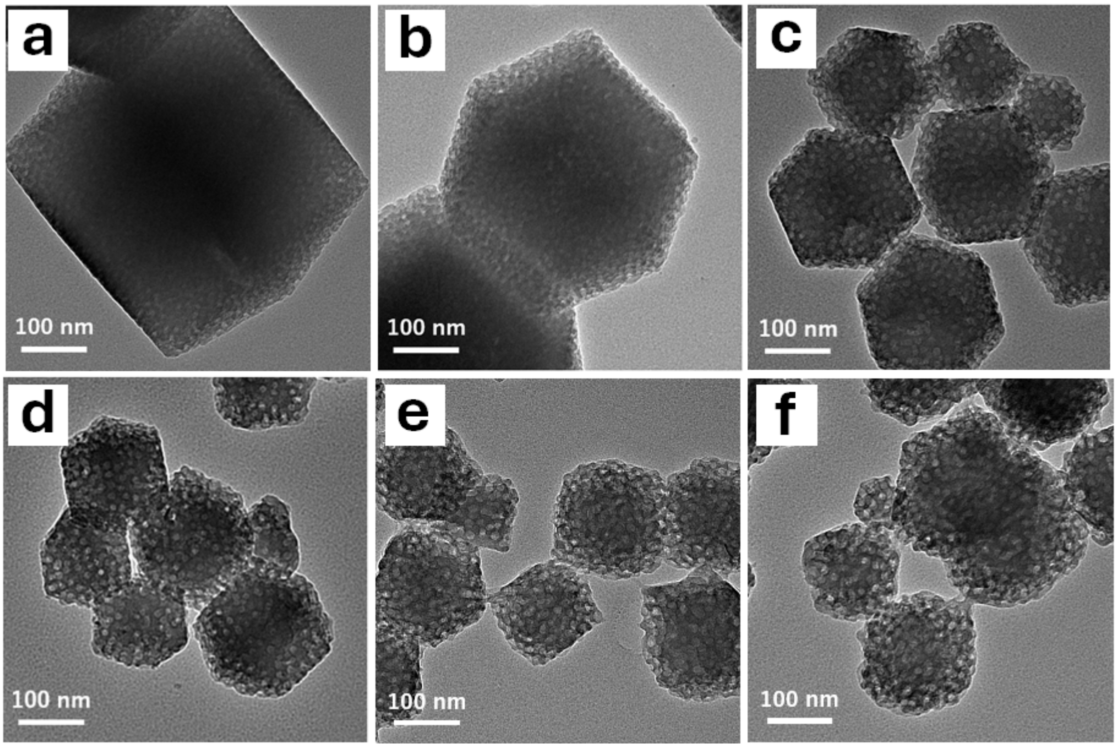

Figure S4 TEM images of X-mZIF-8 synthesized at different temperatures: (a) 0 °C, (b) 20 °C, (c) 40 °C, (d) 50 °C, (e) 60 °C, and (f) 80 °C.

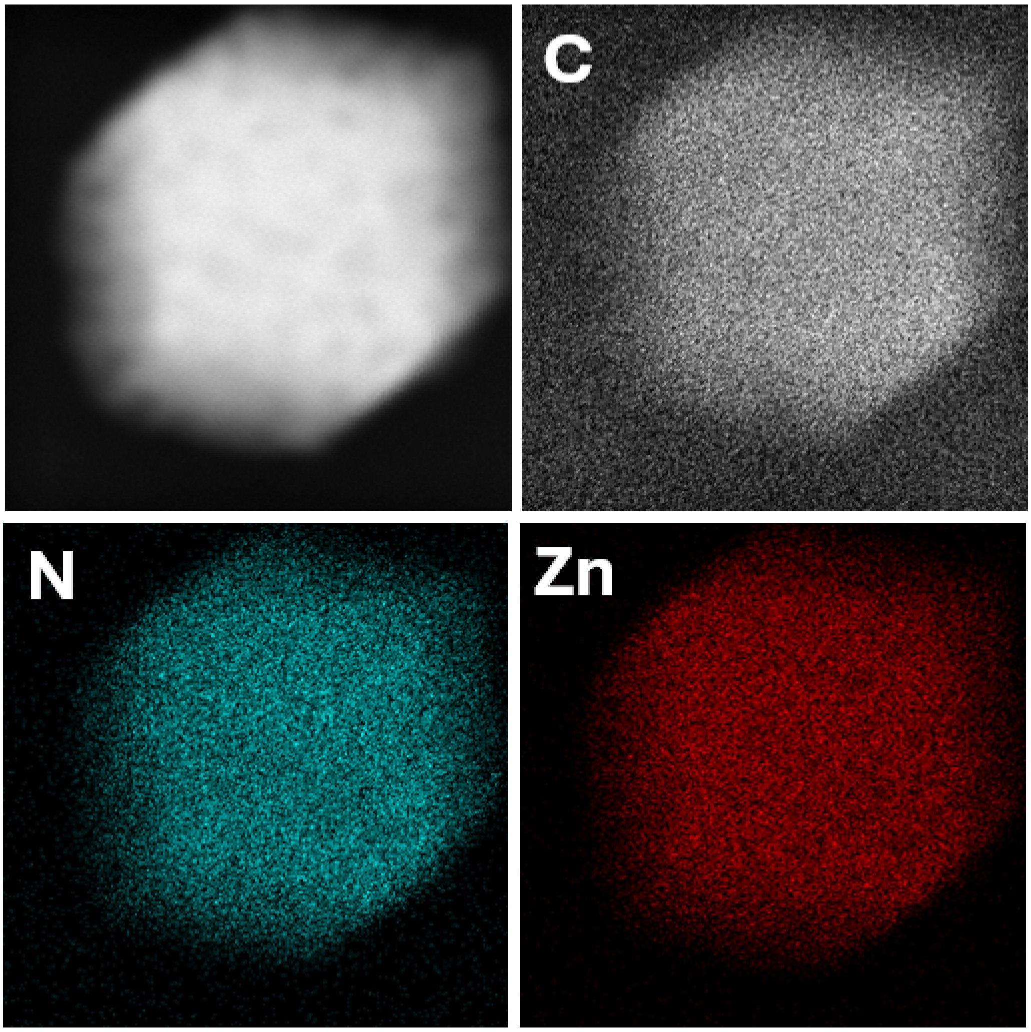

Figure S5 STEM-EDS elemental mapping images of 50-mZIF-8.

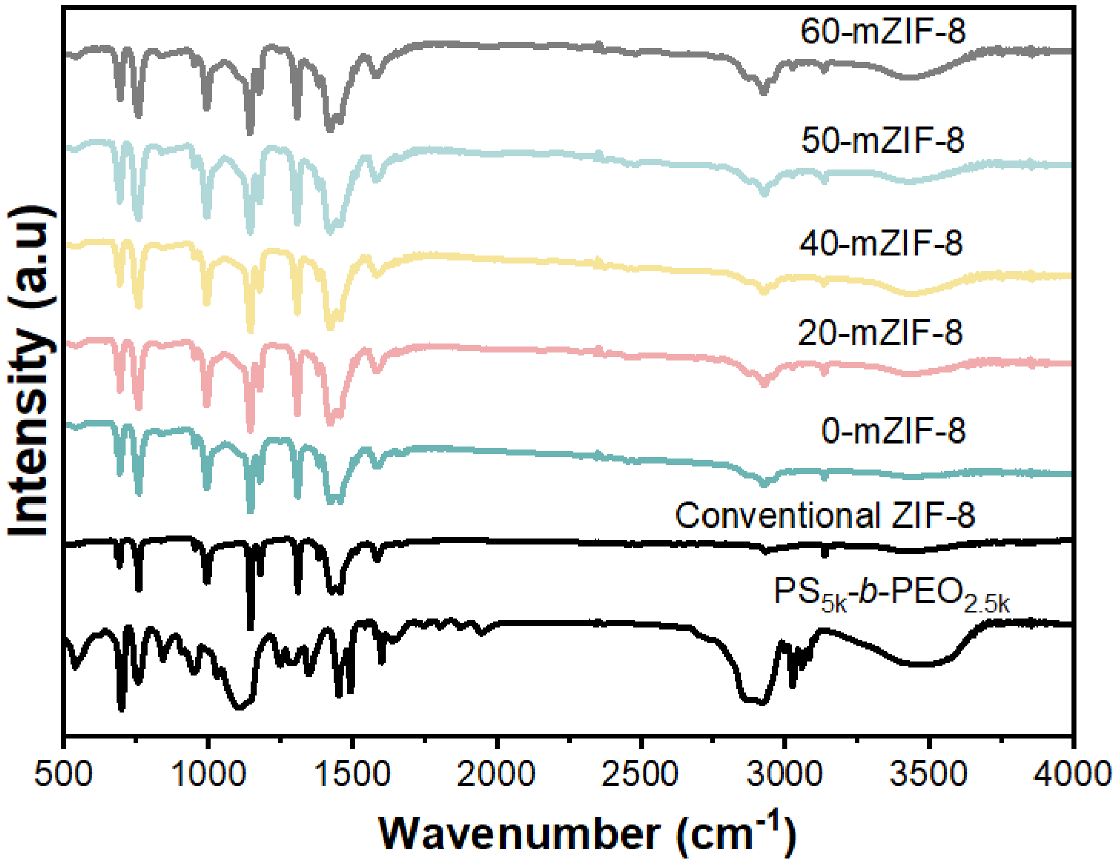

Figure S6 FT-IR spectra of *X*-mZIF-8.

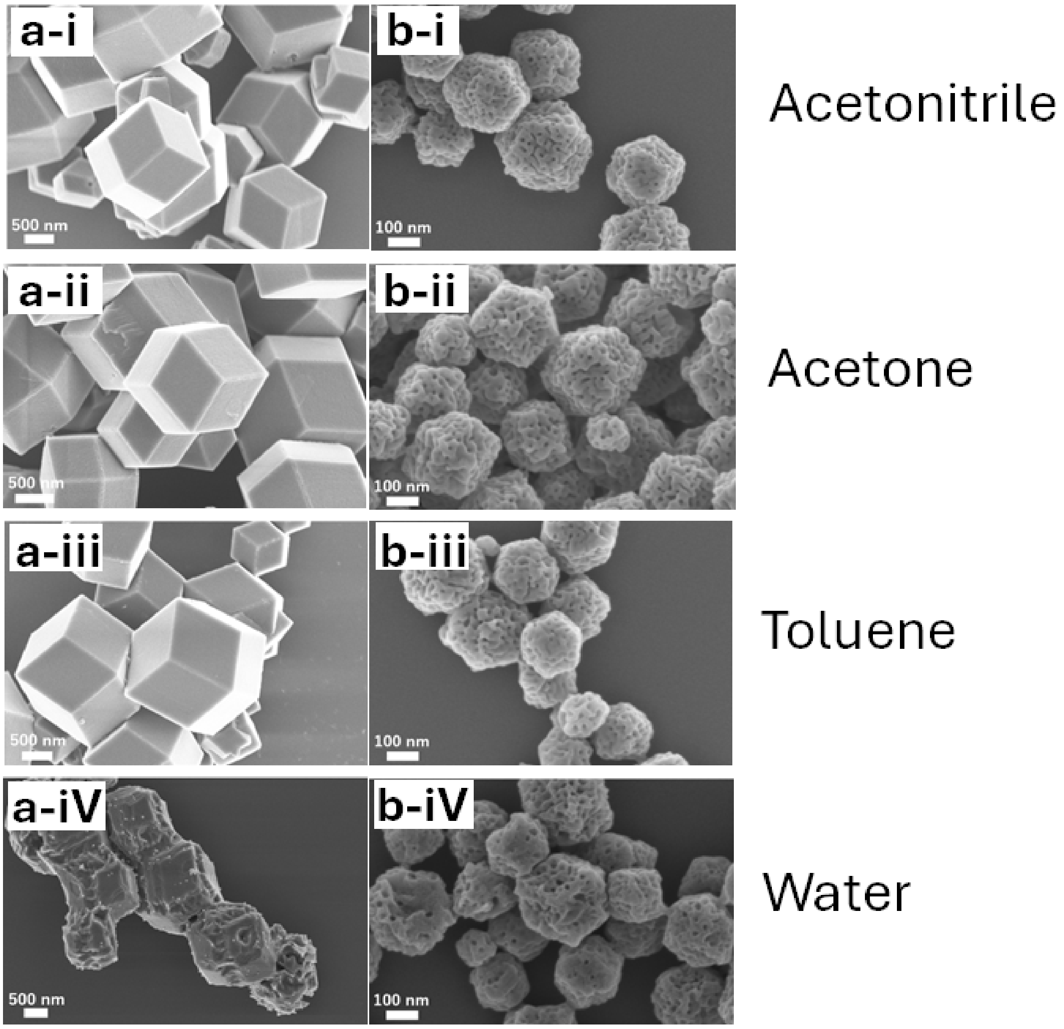

Figure S7 SEM images showing the chemical stability of (a) conventional ZIF-8 and (b) 60-mZIF-8 after exposure to (i) acetonitrile, (ii) acetone, (iii) toluene, and (iv) water, respectively.

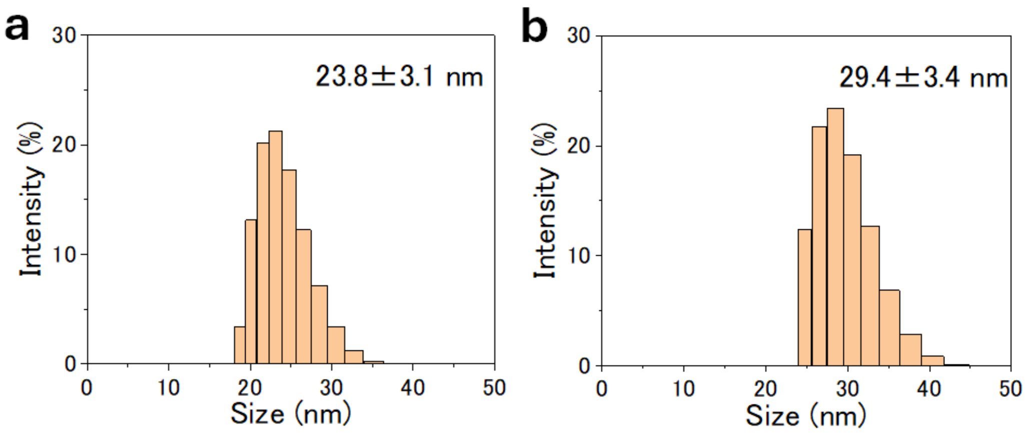

Figure S8 Micelle size distributions at (a) 0 °C and (b) 60 °C obtained from DLS measurements.

Note for **Figure S8** : The sizes measured by DLS include both the hydrophobic and hydrophilic segments of the micelles. Therefore, these values do not correspond directly to the pore sizes of 0-mZIF-8 and 60-mZIF-8.

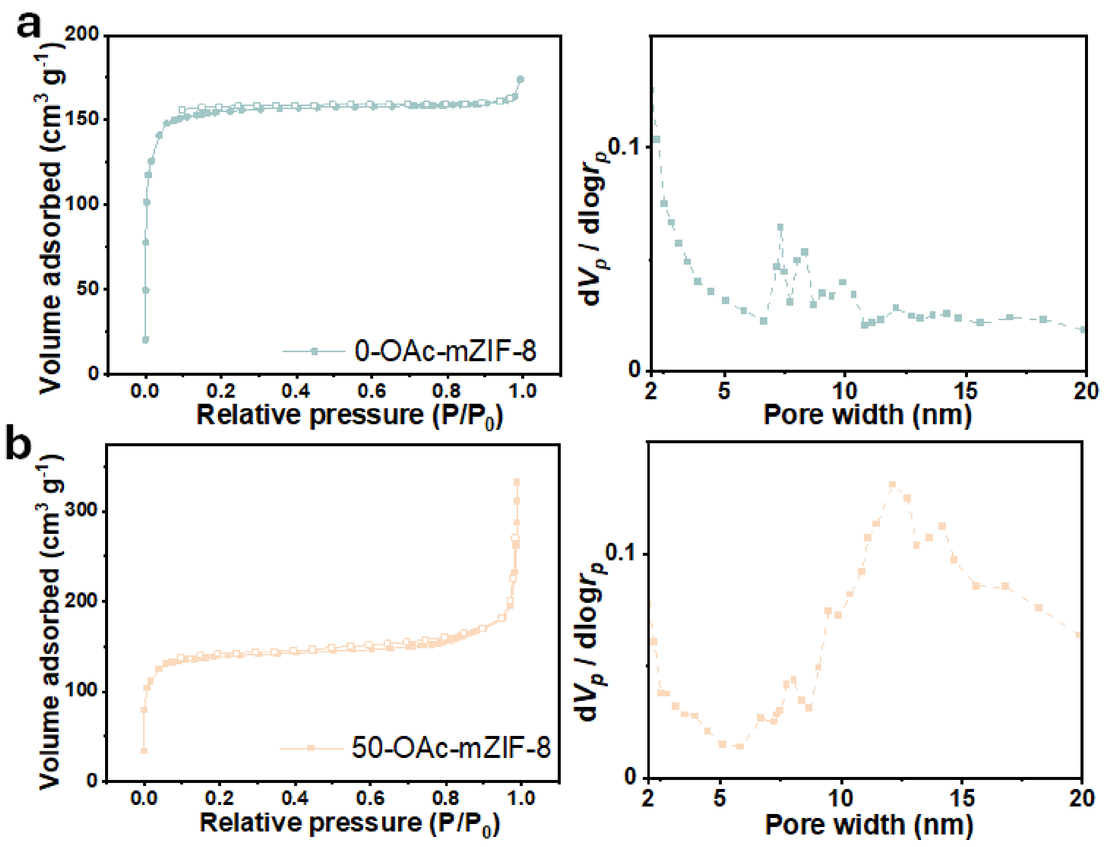

Figure S9 Nitrogen adsorption–desorption isotherms and BJH pore size distributions of (a) 0-OAc-mZIF-8 and (b) 50-OAc-mZIF-8, respectively.

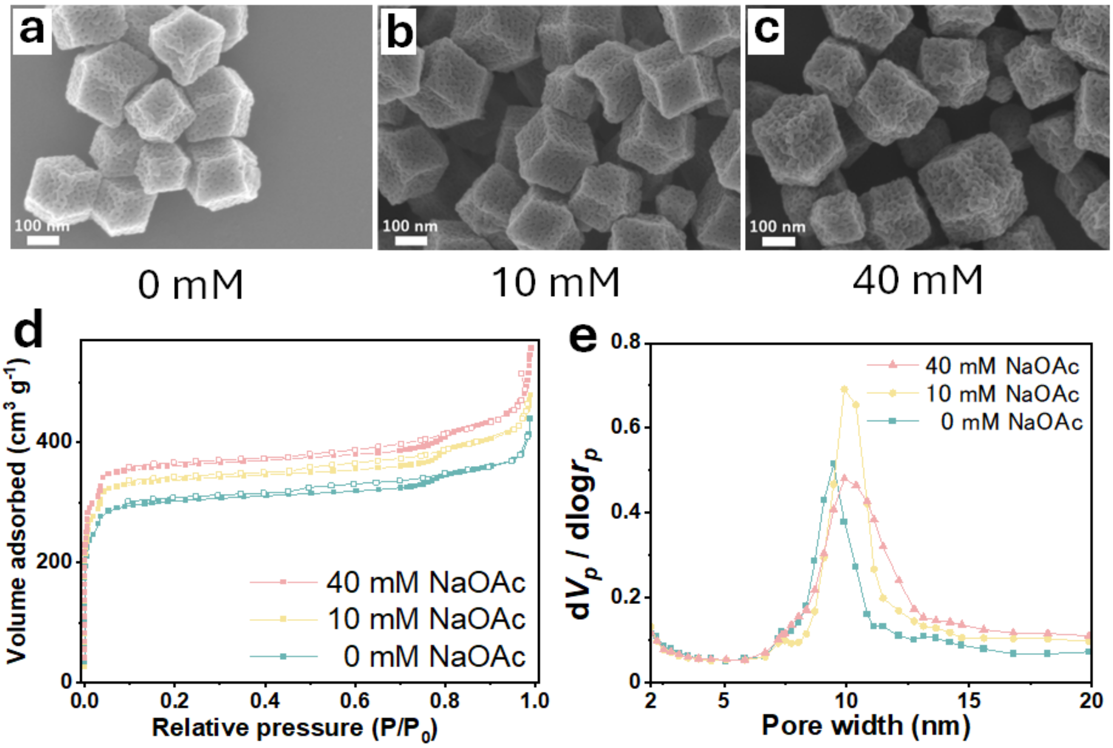

Figure S10 SEM image of mZIF-8 synthesized with (a) 0 mM NaOAc, (b) 10 mM NaOAc and (c) 40 mM NaOAc. (d) Nitrogen adsorption-desorption isotherms and (e) BJH pore size distributions of mZIF-8 synthesized with 0 mM NaOAc, 10 mM NaOAc and 40 mM NaOAc, respectively.

Note for **Figure S10** : To improve the visibility of the nitrogen adsorption-desorption isotherms, an offset of 30 cm^3 g^{-1} and 15 cm^3 g^{-1} in adsorbed volume was applied to the 40 mM and 10 mM NaOAc samples, respectively.

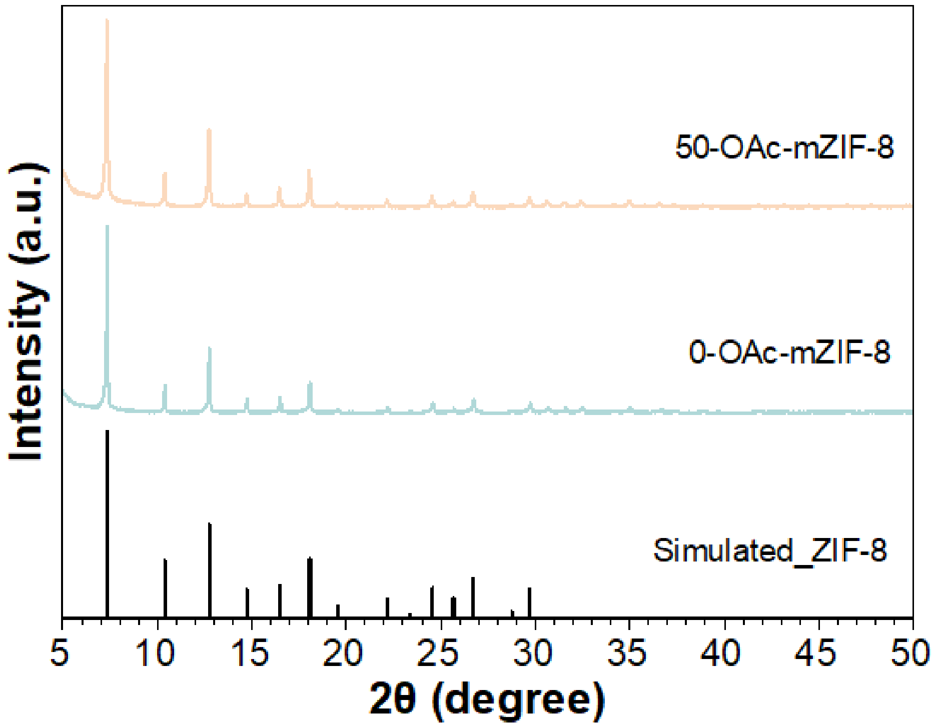

Figure S11 XRD pattern of *X*-OAc-mZIF-8.

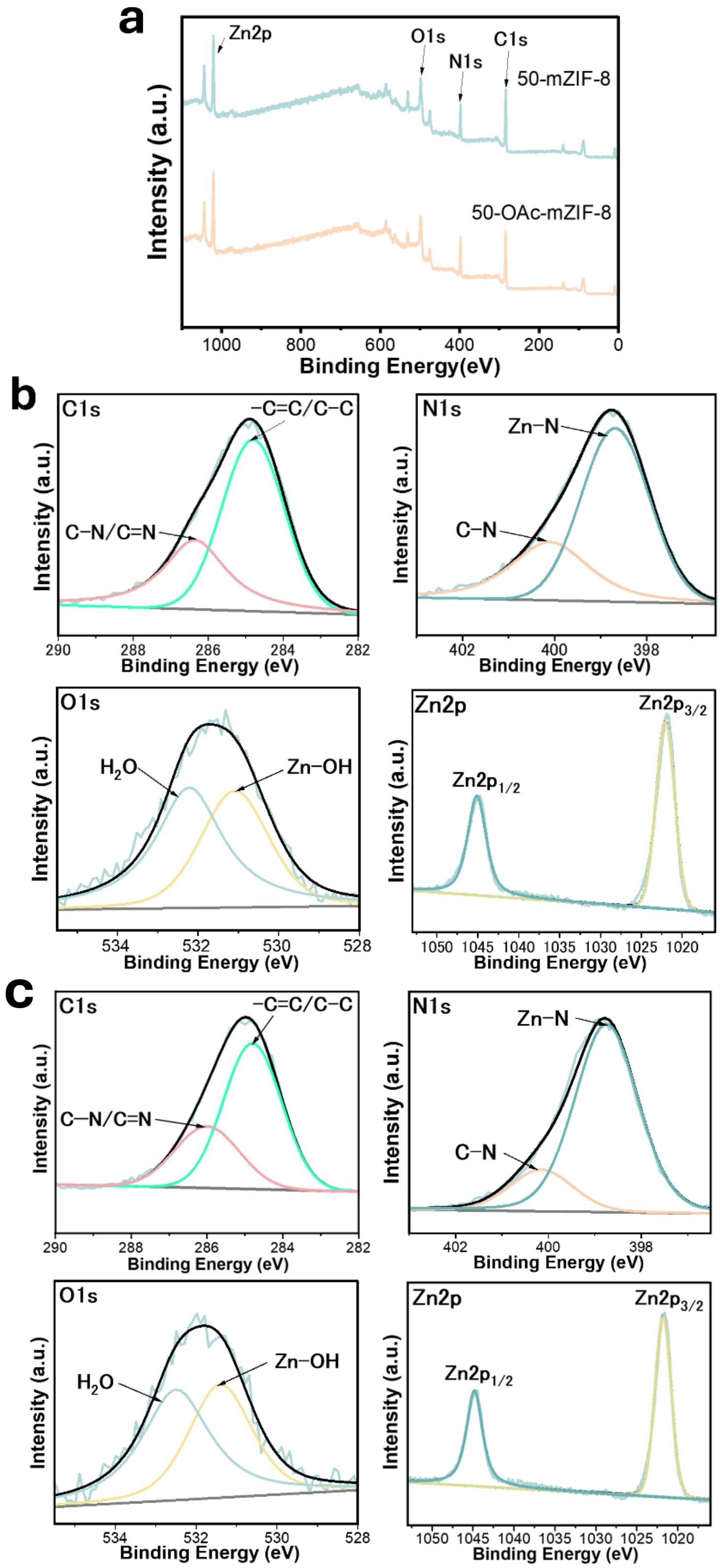

Figure S12 (a) XPS survey spectra of 50-mZIF-8 and 50-OAc-mZIF-8, and high-resolution spectra of (b) 50-mZIF-8 and (c) 50-OAc-mZIF-8.

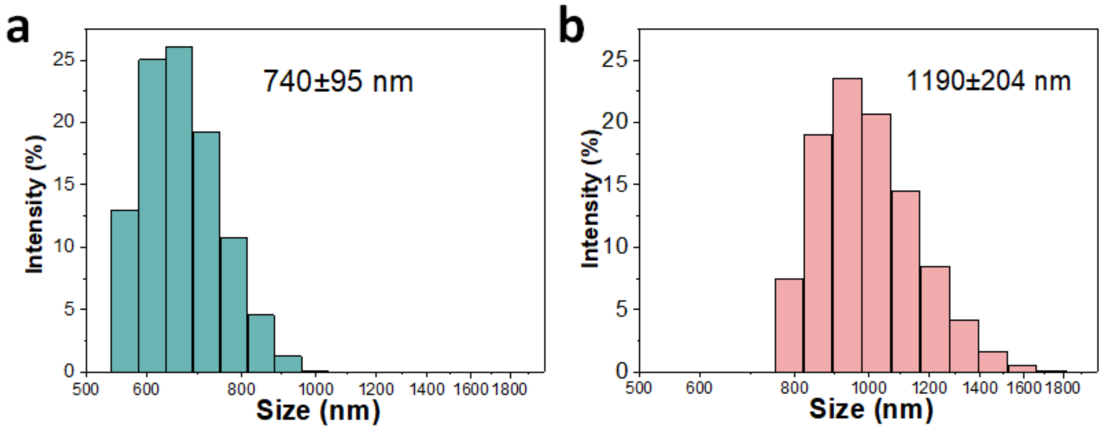

Figure S13 Particle size distributions of (a) 0-OAc-mZIF-8 and (b) TMP-OAc-mZIF-8 obtained from DLS measurements.

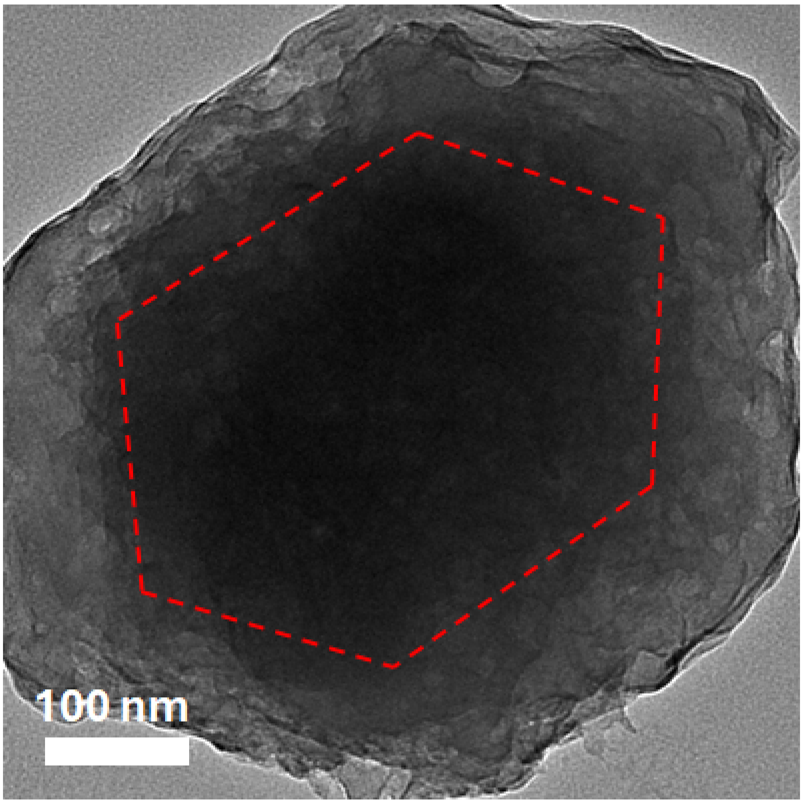

Figure S14 TEM image of TMP-OAc-mZIF-8

Note for **Figure S14** : To obtain a clear TEM image, a smaller-sized particle was intentionally selected, as larger particles prevent electron transmission.

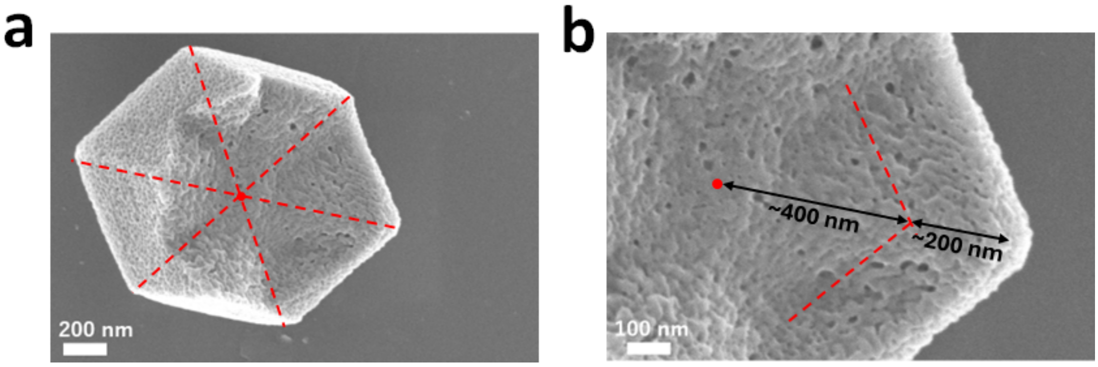

Figure S15 SEM image of (a) Cross-sectional SEM images of TMP-mZIF-8 showing the center point. (b) Cross-sectional SEM image of TMP-mZIF-8 with length of small mesopore-rageon and large mesopore-rageon.

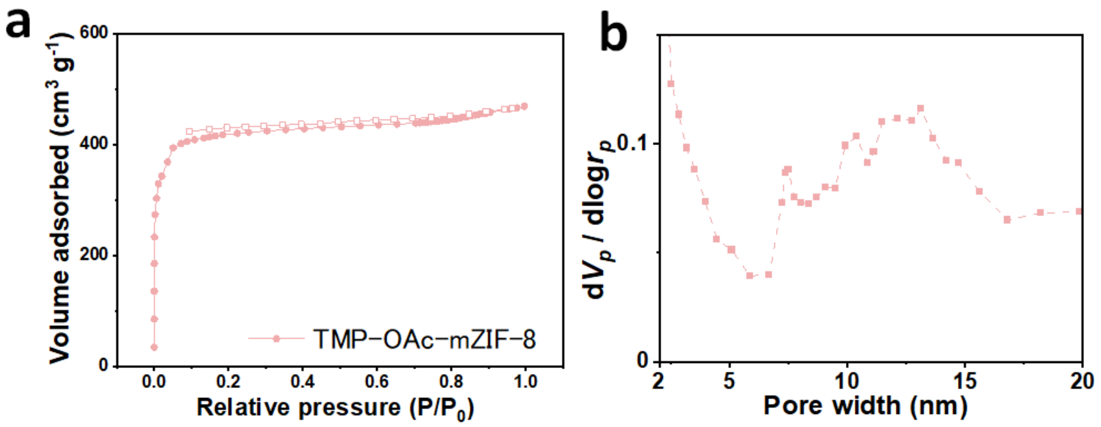

Figure S16 (a) Nitrogen adsorption-desorption isotherm of TMP-OAc-mZIF-8 and (b) BJH pore size distributions of TMP-OAc-mZIF-8, 0-OAc-mZIF-8, and 50-OAc-mZIF-8.
